# The Effects of Short-Term Post-Exposure to 3.6 GHz 5G Mobile Phone Electromagnetic Exposure on Static and Dynamic Postural Control in Healthy Adults: A Randomized Sham-Controlled Pilot Study

**DOI:** 10.3390/s26123750

**Published:** 2026-06-12

**Authors:** Azadeh Torkan, Maryam Zoghi, Negin Foroughimehr, Shapour Jaberzadeh

**Affiliations:** 1 Monash Neuromodulation Research Unit, Department of Physiotherapy, School of Primary and Allied Health Care, Monash University, Melbourne, VIC 3800, Australia; shapour.jaberzadeh@monash.edu; 2 Discipline of Physiotherapy, Institute of Health and Wellbeing, Federation University, Melbourne, VIC 3350, Australia; m.zoghi@federation.edu.au; 3 6G Research and Innovation Lab, Swinburne University of Technology, Hawthorn, Melbourne, VIC 3122, Australia; nforoughimehr@swin.edu.au

**Keywords:** 5G, postural balance, electromagnetic fields, computerized dynamic posturography, limits of stability, sensorimotor integration

## Abstract

The rapid global expansion of 5G technology has increased concerns regarding its potential health effects. Postural balance, a complex sensorimotor function reflecting central nervous system integrity, may be susceptible to electromagnetic field exposure. However, evidence on 5G effects on comprehensive balance outcomes remains limited. This randomized controlled pilot study investigated the effects of short-term exposure to 5G mobile phones on static and dynamic postural balance using computerized posturography. Nineteen healthy adults (mean age: 31 ± 7 years) participated in a randomized crossover design involving three conditions: 5-min exposure, 20-min exposure, and sham. Static and dynamic balance were assessed using the NeuroCom Balance Master, including the Unilateral Stance, Rhythmic Weight Shift, and Limits of Stability tests, which were performed immediately before and after each condition. Two-way repeated-measures ANOVA showed no significant interaction between exposure condition and time (pre vs. post) across all outcomes. Bayesian analyses provided support against detectable exposure-related interaction effects, although evidence for some time-related effects was inconclusive or varied across outcomes. These findings suggest that short-term 5G exposure did not produce detectable alterations in postural control under the experimental conditions tested.

## 1. Introduction

Fifth-generation (5G) mobile phone (MP) technology has rapidly expanded worldwide since its commercial deployment in 2019, increasing public and scientific interest regarding the potential biological effects of radiofrequency electromagnetic fields (RF-EMFs) [1,2,3]. Compared with earlier mobile communication systems, 5G networks operate across broader frequency ranges, including low, mid, and high band frequencies, enabling faster data transfer, reduced latency, and improved connectivity [4,5,6,7,8,9]. Recent developments in open-source and software-defined network architectures have also facilitated practical deployment and experimental implementation of 5G systems under real-world operating conditions [10,11]. Among these, mid-band frequencies (approximately 1–6 GHz) are currently the most widely used for everyday mobile communication because they provide a balance between coverage, signal penetration, and network performance [6,7,8].

MPs emit non-ionizing radiofrequency electromagnetic fields (RF-EMFs), which do not possess sufficient energy to ionize biological tissue [12,13]. Although RF exposure from MPs is regulated within established international safety guidelines [13,14], concerns regarding potential biological effects persist. The increasing proximity, frequency, and duration of MP use have raised questions about whether repeated low-level EM exposure may subtly influence neural systems involved in sensorimotor processing and functional motor control. While previous research has primarily focused on cortical excitability, cognition, sleep, or vestibular physiology, considerably less attention has been directed toward functional motor outcomes such as postural balance.

Postural balance is a complex sensorimotor function that depends on the integration of visual, vestibular, proprioceptive, cerebellar, and cortical systems [15,16,17,18,19,20]. Because balance control requires continuous sensory integration and motor coordination, it may serve as a sensitive functional marker of subtle neurophysiological disturbances. Importantly, many previous studies reporting impaired balance during MP use have attributed these effects primarily to cognitive distraction or dual-task interference associated with texting or speaking, rather than to RF-EMF exposure itself [21,22,23,24]. Consequently, investigations aimed at isolating RF exposure from attentional and behavioral confounders remain limited.

Existing RF-EMF studies have largely focused on vestibular or visual outcomes, as well as measures of brain function and neurophysiology, rather than comprehensive assessments of postural balance [5,25,26,27,28,29,30]. Overall, these studies have generally reported no significant acute disturbances following mobile phone EM exposure. However, objective and comprehensive assessments of postural control using computerized dynamic posturography under controlled real-world 5G mobile phone EM exposure conditions remain scarce.

Unlike isolated neurophysiological or sensory measures, postural balance reflects the integrated function of vestibular, proprioceptive, cerebellar, and cortical systems. Because balance performance depends on coordinated multisystem processing, it may provide a sensitive functional approach for investigating subtle short-term neurophysiological effects of RF-EMF exposure.

To objectively evaluate postural control, the present study used the NeuroCom Balance Master system, a computerized dynamic posturography platform with established validity and reliability for assessing static and dynamic balance performance [31,32,33]. The study focused on three balance domains commonly used in clinical and research settings:

Unilateral Stance (US), which assesses single-leg postural stability and limb-specific balance control [33,34]. Rhythmic Weight Shift (RWS), which evaluates controlled rhythmic weight transfer and dynamic coordination [35,36,37] and Limits of Stability (LOS), which assesses voluntary centre-of-mass displacement and functional balance capacity [38,39,40].

Together, these measures provide complementary information regarding static steadiness, voluntary weight shifting, directional control, and adaptive sensorimotor regulation during both stable and dynamic postural tasks.

Despite growing public concern regarding 5G exposure, evidence regarding its potential influence on functional balance outcomes remains limited. Furthermore, no previous studies have comprehensively examined static and dynamic postural balance using instrumented posturography following controlled short-term predominantly mid-band (~3.6 GHz) 5G mobile-phone exposure under real-world network conditions.

Addressing this gap is important because postural control represents a functionally meaningful outcome reflecting integrated nervous system performance.

This study was motivated by the widespread use of 5G mobile devices and the possibility that localized RF-EMF exposure may influence distributed sensorimotor networks involved in postural regulation. Because postural control depends on integrated multisystem processing, even subtle neurophysiological disturbances could theoretically affect balance performance.

Furthermore, postural control represents a real-world, behaviorally meaningful outcome reflecting the functional integrity of interconnected sensorimotor systems. Even subtle alterations in sensory integration, motor planning, or cortical excitability could theoretically manifest as measurable changes in balance performance. Accordingly, balance assessment may provide a practical functional approach for investigating possible short-term neurophysiological effects of RF-EMF exposure despite the limited penetration depth of mid-band frequencies.

Accordingly, by assessing static, dynamic, unilateral, and rhythmic balance parameters, this study aims to capture functional alterations in postural control that may occur even in the absence of measurable changes in isolated sensory or cortical responses, thereby addressing an important gap in the existing RF-EMF literature.

This study aimed to systematically investigate whether short-duration 5G mobile phone EM exposure affects static and dynamic postural control, addressing a critical gap in the existing literature.

1. To determine whether short-term (5-min) 5G mobile phone EM exposure produces measurable changes in static and dynamic postural outcomes compared with sham conditions.

2. To determine whether 20-min 5G mobile phone EM exposure produces greater alterations in static and dynamic postural outcomes than either the 5-min exposure or sham, indicating potential duration-dependent effects.

We hypothesized that 5G mobile phone EM exposure would produce measurable alterations in postural balance, with greater effects following longer exposure durations.

## 2. Materials and Methods

### 2.1. Participants

Nineteen healthy adults, aged from 18 to 40 (mean ± SD: 31 ± 7 years), completed three conditions in a randomized crossover design: 5-min exposure, 20-min exposure, and sham exposure. Static balance (US) and dynamic balance (RWS, LOS) were assessed using the NeuroCom Balance Master. As this was an exploratory pilot study, the sample was not powered for subgroup analyses. The study primarily focused on feasibility and methodological refinement rather than detailed subgroup comparisons by age or sex. Such analyses may be explored in future studies with larger participant cohorts. The study received ethical approval from the Monash University Human Research Ethics Committee (Approval No. 34411, December 2022), and all participants provided written informed consent in line with the Declaration of Helsinki. Throughout the study, participants were closely monitored for any signs of discomfort or adverse effects, both during and after each session, and were encouraged to report any delayed or lingering reactions.

### 2.2. Sample Size Justification

This research was designed as a pilot study, primarily aimed at evaluating the feasibility of the protocol, refining experimental procedures, and generating preliminary data to guide future, larger-scale investigations [41,42]. Consequently, the sample size was not determined through a formal a priori power calculation. Instead, a pragmatic approach was taken, with 19 participants recruited based on feasibility and available resources, consistent with recommendations for pilot studies [43]. Pilot studies typically enrol between 12 and 30 participants [43,44] to provide sufficient preliminary insight. The results obtained in this study may assist future sample-size calculations and the design of adequately powered confirmatory studies [43,44].

### 2.3. Inclusion-Exclusion Criteria

Eligible participants were healthy, right-handed adults aged 18–40 years, with no known neurological, psychiatric, or musculoskeletal disorders based on a standardized safety screening questionnaire [45]. Individuals were excluded if they reported a history of head trauma, epilepsy in a first-degree relative, metallic objects in the cranial area (other than standard dental work), implanted cardiac devices, neurostimulators, surgical clips, medical pumps, or any other electronic implants. People who experienced recurrent or severe headaches or migraines, those with neurological or psychiatric conditions, individuals taking neuroactive medications, and those who were pregnant were also not eligible.

To minimize confounding factors, participants were instructed to follow several pre-session guidelines: get a minimum of seven hours of sleep, avoid alcohol for 48 h, skip caffeine and energy drinks for three hours, and refrain from vigorous physical activity for 24 h leading up to the testing session. All participants confirmed they had followed these instructions through self-report.

### 2.4. Study Design

A randomized, sham-controlled crossover design was implemented, with a minimum 48-h interval between visits to reduce the likelihood of any residual or carryover effects. Each participant completed all three experimental conditions:

5-min and 20-min MP exposure on talking mode and a duration-matched sham (MP in the switched off condition). This approach allowed every participant to act as their own control, helping to minimize inter-individual variability and strengthen within-subject comparisons. To avoid order or learning effects, the sequence of the three conditions was counterbalanced and randomized for each participant. During each session, participants remained seated comfortably and held the MP in their left hand approximately one centimeter from the left ear, following established procedures used in MP exposure research. All experimental sessions took place between 12:00 and 17:00, a time window selected to reduce the influence of circadian fluctuations on cortical excitability, which are known to vary across the day.

To balance ecological validity and experimental control, participants engaged in a scripted verbal exchange with the experimenter, who sat about two meters behind them, during all three conditions. The dialogue consisted of a brief, standardized list of questions and responses that was identical across sessions. The phone remained muted, so the only audible sound was the participant’s own speech, ensuring that acoustic intensity and dB(A) levels were consistent across the exposure and sham conditions. Although a scripted verbal exchange was included during all sessions, balance assessments were intentionally performed immediately after exposure rather than during active MP operation because pilot testing indicated that active electromagnetic fields (EMFs) interfered with the sensitive force-plate sensors of the NeuroCom Balance Master system. Consequently, the present design could not capture potential transient neuromotor effects occurring exclusively during active exposure.

Balance assessments were conducted after the exposure period rather than during speaking, thereby minimizing any influence of speech-related respiratory mechanics, attentional load, or postural adjustments on balance outcomes. The scripted nature and identical duration of verbal interaction across all conditions further reduced variability attributable to speech.

**Figure 1:**
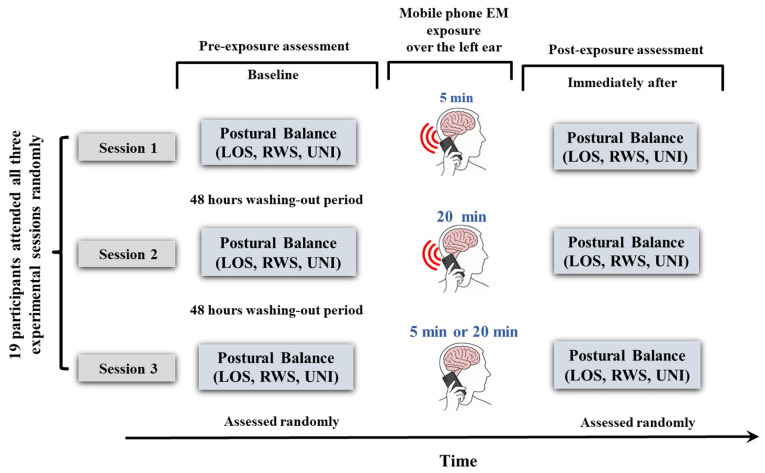
Overview of the randomized, sham-controlled, crossover experimental protocol using NeuroCom Balance Master. (Natus Medical Inc., Pleasanton, CA, USA). Balance outcomes included: Limits of Stability (LOS), Rhythmic Weight Shift (RWS), and Unilateral Stance (US). Red wave symbols indicate active mobile phone electromagnetic exposure, while arrows indicate the progression of time and study procedures.

In the sham condition, the entire procedure was replicated in full, except that the MP was switched off to eliminate radiofrequency output. This design allowed the sham session to serve as a valid control for non-RF factors, including holding the device, posture, and conversational activity, while isolating the effects of EMF exposure (Figure 1).

The 5G MP used in this study was a Samsung Galaxy A13 (Model SM-A136B) manufactured by Samsung Electronics Co., Ltd., South Korea.

The same device was employed in all three experimental conditions to maintain consistency. The phone operated in talk mode only, with no data transmission, and calls were placed through Telstra’s network in the Frankston region of southeast Melbourne.

The handset was selected as a representative commercially available 5G device operating predominantly within the mid-band 5G range (~3.6 GHz) under real-world network conditions, as reported by the Radio Frequency National Site Archive (RFNSA). The Narda EMR-300 broadband EMF meter (Narda Safety Test Solutions GmbH, Pfullingen, Germany) was used for electromagnetic field measurements. Because this instrument is a broadband EMF meter, it was not capable of spectrally isolating individual frequency bands or definitively excluding transient network handovers between 5G and LTE signals during phone operation. Accordingly, the exposure condition should be interpreted as a real-world predominantly mid-band 5G mobile-phone exposure environment rather than a pure isolated 3.6 GHz exposure.

Although reliance on a single MP model may limit the generalizability of the findings to other 5G devices, this study aimed to examine the neural and behavioral effects of a well-defined 5G EMF exposure. Using a device with a clearly characterized emission profile enabled a stable and controlled exposure environment and improved the precision of outcome measurements.

Manufacturer- or regulatory-reported specific absorption rate (SAR) values for the selected handset were provided solely as contextual information regarding compliance with established international exposure and safety standards. Direct SAR measurements were not performed during the experimental sessions, and no participant-specific estimation or modelling of local absorbed dose in the head or ear was undertaken. Consequently, the reported SAR values should not be interpreted as direct measures or proxies of individual tissue-level exposure in the present study. Exposure characterization was instead based on experimentally measured environmental RF field parameters, including power density and electric field strength [13,46].

### 2.5. Sham Intervention

During the sham condition, participants held the same MP in the same position as in the active exposure sessions; however, the device was powered off to ensure that no EM signal was emitted. This setup controlled for factors such as hand position, device weight, and tactile sensations. Although the sham condition controlled for device position, weight, posture, and conversational activity, complete participant blinding was not possible because the phone was powered off. Therefore, expectancy or placebo/nocebo effects cannot be fully excluded and should be considered when interpreting the null findings.

### 2.6. Exposure Setting

#### Device Specifications and EMR Compliance

The Samsung Galaxy A13 5G, which operates primarily at approximately 3.6 GHz, was selected because it reflects the performance characteristics of common mid-band 5G devices. According to the Radio Frequency National Site Archive (RFNSA) [47] and monitoring by the Australian Radiation Protection and Nuclear Safety Agency (ARPANSA), electromagnetic emissions at the study location were approximately 2.20% of the public exposure limit. This estimate was based on a distance of 46 m from the relevant transmission facility.

All RF exposure values reported in this study, such as power density (PD = 0.0030 W/m^2^) and electric field strength (E-field = 1.5 V/m), were obtained using direct, real-time laboratory measurements. These measurements reflected environmental RF field levels recorded within the laboratory during active phone operation rather than direct near-field dosimetry estimates at the participant’s head or ear. Direct SAR or local absorbed-dose measurements were not performed. Accordingly, the present findings should be interpreted as reflecting experimentally measured environmental RF exposure conditions rather than precise participant-specific tissue dosimetry. Measurements were taken during active phone operation using a Narda EMR-300 radiation meter (Narda Safety Test Solutions GmbH, Pfullingen, Germany), allowing a precise assessment of exposure conditions independent of RFNSA environmental summaries.

The handset used in the experiment supports multiple 4G and 5G bands, including 0.7 GHz, 0.85 GHz, 1.8 GHz, 2.1 GHz, 2.6 GHz, and 3.6 GHz. In Australia, allocation and regulation of these communication bands fall under the responsibility of the Australian Communications and Media Authority (ACMA) [48].

Low-band 5G (<1 GHz) supports long-distance coverage and strong indoor penetration, albeit with reduced bandwidth. Mid-band 5G (1–6 GHz), which includes the device’s 3.6 GHz band, balances coverage, reliability, and network speed. In contrast, high-band 5G, also referred to as millimeter-wave (mm Wave), operates at frequencies around 26 GHz, enabling very high speeds but offering limited penetration and short range. For these reasons, the study concentrated specifically on predominantly mid-band 5G exposure, as this represents one of the most common exposure conditions associated with contemporary MP use in Australia.

All laboratory measurements were repeated five times and averaged to improve measurement reliability. Background EMR readings were collected under two conditions: first, with all nearby devices powered off, and then with all devices active, to screen for unintended EM interference (EMI). These procedures followed recommendations from the International Commission on Non-Ionizing Radiation Protection (ICNIRP) for environmental EMF assessment.

Throughout each experimental session, real-time PD and E-field values were recorded to ensure accurate monitoring of exposure. All measured levels were well below internationally accepted safety thresholds. ICNIRP [13] and IEEE [49] guidelines specify a maximum public exposure limit of 40 W/m^2^ for PD within the 2–6 GHz range. In contrast, during the active mobile-phone condition, the highest observed PD was 0.0030 W/m^2^, and the measured E-field values (average 1.5 V/m, calculated 1.06 V/m) remained substantially lower than these safety limits.

For context, ARPANSA’s Environmental Electromagnetic Energy (EME) reporting indicates typical base-station levels of 2.4 V/m and 0.01644 W/m^2^ at distances of 400–500 m from a transmitter. The comparatively lower readings in our study likely reflect differences in operational conditions rather than measurement error. ARPANSA reports the maximum possible output assuming all transmitters at a site are active and running at full power [14], whereas in real settings, many transmitters remain off until call or data demand increases. Modern networks also regulate handset power dynamically, reducing transmission strength when signal quality is high or when the device is close to the base station. 

### 2.7. Outcome Measures

Postural control was assessed using the NeuroCom Balance Master system (Natus Medical Incorporated). All balance assessments were conducted in the same quiet laboratory environment under controlled lighting (300–350 lux) and stable ambient temperature (22–23 °C), with no external noise sources present. The NeuroCom system was positioned on a level, vibration-free floor to ensure consistency and reproducibility of measurements [31]. The following protocols were administered:

#### 2.7.1. Unilateral Stance Test (US)

Postural stability during single-leg stance was quantified as sway velocity, expressed in degrees per second (deg/s) [31]. 

#### 2.7.2. Rhythmic Weight Shift (RWS)

Dynamic postural control was evaluated by measuring participants’ ability to rhythmically transfer body weight in the left–right and front–back directions. The main RWS outcomes were on-axis velocity (O-AV, deg/s) and directional control (DCL, %) [50].

#### 2.7.3. Limits of Stability (LOS)

Dynamic postural control and voluntary weight-shifting ability were assessed using the LOS test as an individual’s ability to intentionally displace their center of gravity to their theoretical stability limits and maintain stability at those endpoints [51]. During the test, participants were instructed to shift their center of gravity in a controlled manner to move a cursor toward eight predetermined targets displayed on the screen, corresponding to forward, backward, left, right, and the four diagonal directions. The test provides quantitative data on several key aspects of motor control, including Reaction Time (RT in sec), defined as the time delay between the command to move and the initiation of movement; Movement Velocity (MVL in deg/s), representing the average speed of the center of gravity movement toward the target; Endpoint Excursion (EPE in %), referring to the distance of the first movement toward the target, indicating initial movement accuracy; Maximum Excursion (MXE in %), defined as the furthest distance the center of gravity travels toward the target, reflecting the functional LOS; and Directional Control (DCL in %), which is a measure of the movement’s straightness, calculated as the amount of on-path movement minus extraneous off-path movement.

### 2.8. Procedure

Participants completed a standardized familiarization phase before formal data collection for all balance protocols. For the US test, participants performed one practice trial for each stance condition to ensure that they understood the task and performed it correctly. For the RWS test, one practice trial was completed for each movement direction (left–right and front–back) to familiarize participants with the visual feedback and task requirements. For the LOS test, two practice trials were conducted to ensure that participants understood the voluntary leaning task and target-directed center-of-gravity movement. For all protocols, standardized written instructions were delivered to all participants, with consistent wording, tone, demonstrations, and the number of practice trials. This approach minimized experimenter-related variability and helped ensure that observed differences in balance performance reflected participant performance or experimental conditions rather than inconsistencies in test administration. Data from the automatically generated NeuroCom PDF reports constituted the primary dataset for balance outcomes in this study.

The US test was performed in the: left leg eyes open (Left-EO), left leg eyes closed (Left-EC), right leg eyes open (Right-EO), and right leg eyes closed (Right-EC). Loss-of-balance duration (LOB, sec) was also recorded for each trial. This test evaluates the ability to maintain stability on a reduced base of support and provides information on visual dependence, proprioceptive control, and inter-limb differences [16]. Lower sway velocity values reflect better postural control, whereas higher sway velocity values reveal greater postural instability, particularly when visual input is removed under eyes-closed conditions. Sway velocity from the US test was included as a primary static balance outcome [52,53,54].

The RWS test was performed in the left–right and front–back directions at slow, moderate, and fast movement speeds. For each direction and speed, the system calculated two primary metrics: on-axis velocity (O-AV, deg/s), reflecting the speed and smoothness of weight-shifting performance, and directional control (DCL, %), indicating how accurately participants stayed within the intended movement path while minimizing extraneous sway. Higher on-axis velocity shows faster coordinated weight transfer, whereas higher directional control indicates more accurate and efficient movement execution. Together, these parameters provide measures of dynamic balance and sensorimotor integration during rhythmic postural adjustments [16], making RWS a robust indicator of functional postural adaptability [55,56].

The LOS test was used to assess voluntary motor control and the functional limits of stability [52]. While viewing a visual display with a central starting position and eight radially arranged targets, participants were instructed to shift their body weight as quickly and accurately as possible to move the center-of-gravity (COG) cursor toward each highlighted target and then return to the center. Participants were instructed not to step, lift their feet, or use external support during the task. The LOS test generated outcome measures for each of the eight directions and composite scores across directions. The analyzed variables were Reaction Time (RT, sec), Movement Velocity (MVL, deg/s), Endpoint Excursion (EPE, %), Maximum Excursion (MXE, %), and Directional Control (DCL, %). Standardized instructions and practice trials were used across all protocols to minimize test-administration variability. 

### 2.9. Statistical Analysis

Statistical analyses were conducted to assess the effects of exposure to 5G mobile phone EM on static and dynamic balance outcomes. A two-way repeated-measures ANOVA was conducted to examine the effects of exposure condition (5-min EM exposure, 20-min EM exposure, and sham), time (Pre and Post), and the exposure condition × time interaction on the primary balance outcomes. Because this study was designed as an exploratory pilot study, the frequentist analyses were interpreted as descriptive and hypothesis-generating rather than confirmatory. These outcomes included US sway velocity, RWS on-axis velocity and directional control, and LOS composite scores. Analyses were performed using GraphPad Prism version 10.1.2 [57]. Outliers were assessed using GraphPad Prism’s ROUT method, with Q set at 1%. Data that deviated from normality were logarithmically transformed before statistical analysis to better satisfy the assumptions of parametric testing. Normality was subsequently assessed using the Shapiro–Wilk test and visual inspection of Q–Q plots. All statistical analyses were conducted in a blinded manner with respect to exposure condition. Complete participant blinding could not be guaranteed because participants may have been aware of whether the MP was powered on or off; however, they were not informed of the specific study hypotheses or the expected direction of any effects. The investigator administering the balance assessments was aware of the exposure condition, while data processing and statistical analyses were performed by an investigator blinded to condition allocation. This partial blinding approach minimized analytical bias while acknowledging the practical constraints of the exposure protocol. Post hoc comparisons were performed for significant main effects or interactions using Tukey’s multiple comparisons test to control the family-wise error rate. This adjustment accounts for the number of comparisons within each test family, yielding adjusted *p*-values. Given the exploratory pilot-study design and the number of balance outcomes assessed across the US, RWS, and LOS domains, *p*-values were interpreted cautiously and considered alongside Bayesian evidence rather than as definitive evidence of exposure-related effects.

To complement the frequentist analyses and quantify the strength of evidence, Bayesian repeated-measures ANOVAs were conducted using JASP version 0.19.3 [58]. Bayes factors (BF_10_) were calculated to compare the relative support for models including exposure condition, time, and their interaction against the null model. According to conventional interpretation [59,60], BF_10_ values of 1–3 were considered anecdotal evidence, 3–10 moderate evidence, 10–30 strong evidence, 30–100 very strong evidence, and >100 extreme evidence for the alternative hypothesis. BF_10_ values close to 1 were interpreted as inconclusive, whereas values below 1 were interpreted as increasingly favoring the null hypothesis as they approached zero.

## 3. Results

### 3.1. Static Balance: Unilateral Stance (US)

US performance was assessed under eyes-closed (US-EC) and eyes-open (US-EO) conditions. Overall, 5G mobile phone EM exposure did not produce detectable changes in static postural control.

**Figure 2:**
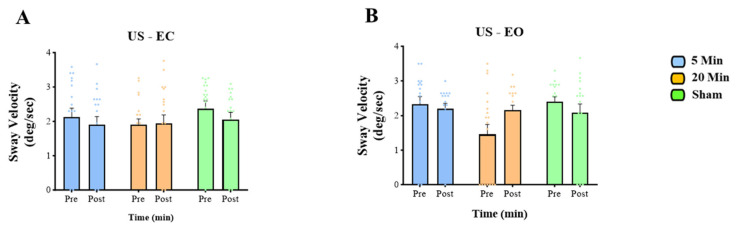
Sway velocity (deg/s) during the Unilateral Stance (US) test across exposure conditions (5-min, 20-min, and sham), measured before (Pre) and after (Post) 5G mobile phone RF-EMF exposure (**A**) Eyes-closed condition (US-EC); (**B**) Eyes-open condition (US-EO). Blue bars represent the 5-min exposure condition, orange bars represent the 20-min exposure condition, and green bars represent the sham condition. For visualization, difference scores were calculated as Post–Pre, with positive values indicating increased sway velocity and reduced postural stability, and negative values indicating decreased sway velocity and improved postural stability. Data are presented as mean ± standard error, with individual participant values overlaid. No significant Exposure Condition × Time interactions were observed for either US-EC or US-EO outcomes (all *p* > 0.79), and Bayesian analyses supported the absence of detectable exposure-related interaction effects (BF_10_ range = 0.013–0.048).

Static balance performance during the US test was not detectably altered by 5G mobile phone EM exposure. For both US-EC and US-EO sway velocity outcomes, no Exposure × Time interactions or main effects of Exposure Condition were observed (all *p* > 0.29). Bayesian analyses similarly supported the absence of detectable exposure-related interaction effects across US outcomes (BF_10_ range = 0.013–0.048) (Figure 2).

Collectively, these findings indicate that unilateral stance performance and static postural stability were not detectably altered following either 5-min or 20-min 5G mobile phone EM exposure compared with sham. 

### 3.2. Dynamic Balance: Rhythmic Weight Shifting (RWS)

**Figure 3:**
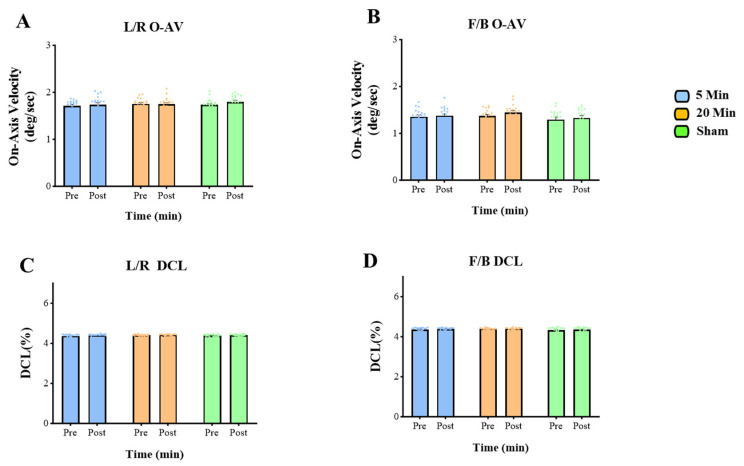
Rhythmic Weight Shift (RWS) performance across exposure conditions (5-min, 20-min, and sham), measured before (Pre) and after (Post) 5G mobile phone EM exposure. Outcomes include on-axis velocity (O-AV, deg/s) for (**A**) left–right and (**B**) front–back movements, and directional control (DCL, %) for (**C**) left–right and (**D**) front–back movements. Higher O-AV values indicate faster coordinated weight shifting, whereas higher DCL values indicate more accurate movement along the intended path. Data are presented as mean ± standard error, with individual participant values overlaid. No significant Exposure Condition × Time interactions or main effects of Exposure Condition were observed across RWS outcomes (all *p* > 0.24). Bayesian analyses also supported the absence of detectable exposure-related interaction effects (BF_10_ range = 0.031–0.036).

Dynamic balance performance during the RWS test was not detectably altered by 5G mobile phone EM exposure. For left–right and front–back on-axis velocity (O-AV), no Exposure × Time interactions or main effects of Exposure condition were observed (all *p* > 0.24). Bayesian analyses supported the absence of detectable exposure-related interaction effects for O-AV outcomes (BF_10_ range = 0.031–0.036). Directional control (DCL) measures in both movement planes also showed no Exposure × Time interactions and no main effects of Exposure condition (all *p* > 0.33). A small, exposure-independent improvement over time was observed for front–back O-AV. However, Bayesian analysis indicated that evidence for the Time effect remained inconclusive, while model comparisons generally favored models without exposure-related effects (Figure 3).

Collectively, these findings indicate that rhythmic weight-shifting velocity and directional control remained stable following exposure to 5G mobile phones.

### 3.3. Dynamic Balance: Limits of Stability (LOS)

**Figure 4:**
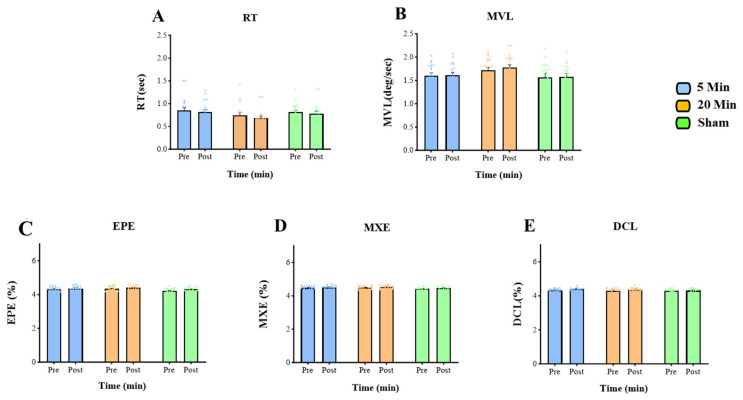
Limits of Stability (LOS) performance across exposure conditions (5-min, 20-min, and sham), measured before (Pre) and after (Post) 5G mobile phone EM exposure. Outcomes include (**A**) Reaction Time (RT, sec), (**B**) Movement Velocity (MVL, deg/s), (**C**) Endpoint Excursion (EPE, %), (**D**) Maximum Excursion (MXE, %), and (**E**) Directional Control (DCL, %). Lower RT values indicate faster movement initiation, whereas higher MVL, EPE, MXE, and DCL values indicate better LOS performance. Data are presented as mean ± standard error, with individual participant values overlaid. No significant Exposure Condition × Time interactions were observed across LOS outcomes (all *p* > 0.10). Bayesian analyses generally supported the absence of detectable exposure-related interaction effects (BF_10_ range = 0.093–0.147).

Limits of Stability (LOS) outcomes showed no evidence of exposure-related changes across composite measures. Reaction time (RT) and movement velocity (MVL) showed no Exposure × Time interactions and no main effects of Exposure condition (all *p* > 0.10). Bayesian analyses supported the absence of detectable Exposure × Time interaction effects for these outcomes (BF_10_ range = 0.093–0.147). Endpoint excursion (EPE), maximum excursion (MXE), and directional control (DCL) also showed no Exposure × Time interactions. Small main effects of Time were observed for EPE, MXE, and DCL (*p* < 0.05), reflecting modest Pre-to-Post improvements that were not specific to any exposure condition. Bayesian analyses indicated that evidence for some Time effects was variable and, in several cases, remained inconclusive, but did not support exposure-related interaction effects (Figure 4).

Overall, LOS performance changes were exposure-independent and may reflect task familiarization or learning effects rather than neuromotor effects of 5G mobile phone EM exposure.

### 3.4. Summary of Results

Across static and dynamic balance domains, including unilateral stance, rhythmic weight shift, and limits of stability, no Exposure × Time interactions were observed. These findings indicate no detectable exposure-related changes under the tested experimental conditions, although subtle effects cannot be completely excluded given the exploratory pilot design and modest sample size. Small improvements over time were observed for selected RWS and LOS outcomes, likely reflecting task familiarization or practice effects rather than exposure-related changes. Bayesian analyses also supported the absence of detectable exposure-related interaction effects across balance outcomes.

To complement the graphical presentation of the findings and improve transparency of data distribution and variability, descriptive statistics (mean ± SD) for the primary balance outcomes across all exposure conditions are presented in Table 1, Table 2 and Table 3. All repeated-measures ANOVA and Bayesian analyses were conducted using log-transformed values rather than raw measurements. Negative values observed for some variables, such as RT-Composite, reflect the mathematical properties of the logarithmic transformation and do not indicate negative reaction times.

## 4. Discussion

This randomized, sham-controlled pilot study examined whether short-term mid-band 5G mobile phone EM exposure influences static and dynamic postural control in healthy adults. Across US, RWS, and LOS outcomes, no Exposure × Time interactions were observed following either 5-min or 20-min exposure compared with sham. Bayesian analyses generally supported the absence of detectable exposure-related interaction effects, although small time-related improvements were observed for selected dynamic balance outcomes. Together, these findings are consistent with the interpretation that brief, low-intensity, predominantly mid-band (~3.6 GHz) 5G mobile-phone EM exposure did not detectably disrupt postural control in healthy adults under the tested conditions. The exposure environment was characterized by low-level environmental RF measurements recorded within the laboratory setting (PD ≈ 0.003 W/m^2^; E-field ≈ 1.5 V/m) under real-world network conditions.

### 4.1. Effect of 5G Mobile Phone EM Exposure on Static Balance: US

We hypothesized that 5- and 20-min 5G mobile phone EM exposure might influence single-leg postural steadiness through possible effects on vestibular or proprioceptive pathways. However, neither the US–Eyes Open nor the US–Eyes Closed condition showed an Exposure × Time interaction. Sway velocity remained stable across exposure conditions, indicating no detectable exposure-related change in static postural control.

Bayesian analyses provided strong support for the absence of detectable exposure-related interaction effects (US-EC BF_10_ = 0.013; US-EO BF_10_ = 0.048). Although very small effects cannot be definitively excluded, these findings suggest that moderate or large exposure-related effects on static postural steadiness are unlikely under the tested conditions.

These results align with earlier RF research demonstrating minimal vestibular effects during acute exposure [25,26,61]. However, they contrast with some studies reporting sway changes during GSM MP exposure [62,63]. This discrepancy may stem from methodological differences; prior positive reports often used non-instrumented tests, uncontrolled handset distance, or unclear exposure profiles. In contrast, the present study used the NeuroCom system and a controlled, dosimetrically characterized exposure setup, strengthening the interpretation of the null exposure-related findings. From a biophysical perspective, the mid-band 5G frequency used in this study (3.6 GHz) has a shallow penetration depth, making direct interaction with deep vestibular or proprioceptive structures unlikely at the low exposure intensity applied (0.0030 W/m^2^). Accordingly, the rationale for examining postural balance in the present study was based primarily on the possibility of subtle systems-level sensorimotor effects rather than direct vestibular tissue penetration.

Thus, short-term 5G mobile phone EM exposure did not detectably impair static postural control in healthy adults under the tested conditions.

### 4.2. Effect of 5G Mobile Phone EM Exposure on Dynamic Balance: RWS

Dynamic balance outcomes during RWS, including O-AV and DCL, did not show detectable exposure-related changes following 5G mobile phone EM exposure. Both left–right and front–back RWS outcomes showed no significant Exposure × Time interaction. Bayesian analyses supported the absence of detectable exposure-related interaction effects for these measures (e.g., L/R O-AV BF_10_ = 0.031; F/B O-AV BF_10_ = 0.036). Although very small effects cannot be definitively excluded, these findings suggest that moderate or large exposure-related effects on rhythmic dynamic balance are unlikely under the tested conditions.

These findings align with studies showing that RF-EMF does not disrupt visuomotor integration or movement timing, mechanisms essential for rhythmic postural adjustments [64,65,66,67,68,69]. Importantly, real-world balance impairments during smartphone use may arise from dual-task cognitive distraction rather than EM exposure itself [70,71,72]. The present study isolated the EMF component under controlled conditions and found no evidence that short-term 5G mobile phone EM exposure reproduced the dynamic balance deficits reported during active phone use.

### 4.3. Effect of 5G Mobile Phone EM Exposure on Dynamic Balance: LOS

LOS metrics, including RT, MVL, EPE, MXE, and DCL, showed no detectable exposure-related changes across conditions. No Exposure × Time interaction was observed, and Bayesian analyses generally supported the absence of detectable exposure-related interaction effects, with BF_10_ values < 1 across LOS parameters (Samsung Galaxy A13, Telstra 5G, 3.6 GHz; PD ≈ 0.003 W/m^2^, E ≈ 1.5 V/m; <ICNIRP/IEEE limits).

Given that LOS performance relies on integrated visual, vestibular, somatosensory, and cognitive control, the absence of measurable exposure-related changes suggests that short-term 5G mobile phone EM exposure did not detectably impair voluntary postural regulation under the tested conditions.

Previous studies examining RF-EMF exposure from MPs similarly suggest no disruption to vestibular pathways or multisensory integration [26,62,73,74]. The use of validated computerized dynamic posturography in the present study further strengthens the interpretation of the null exposure-related findings.

### 4.4. Synthesis of Balance Domain Findings

A key strength of this study is the consistent pattern of null exposure-related findings across functionally distinct balance domains. The absence of detectable effects on static (US), rhythmic dynamic (RWS), and goal-directed voluntary (LOS) postural control suggests that 5G mobile phone EM exposure, under the exposure parameters tested in this study, did not disrupt reflexive stabilization, visuomotor coordination, or integrated sensorimotor planning.

The convergence of findings across these domains strengthens the overall interpretation that brief 5G mobile phone EM exposure did not produce detectable alterations in postural control in healthy adults under the tested conditions.

### 4.5. Interpretation of Null Findings and Biological Plausibility

Across static and dynamic balance outcomes, the findings showed no detectable exposure-related effects or, for some outcomes, inconclusive evidence for very small effects. Because Bayes factors generally favored the null model or were close to 1, the results should be interpreted as an absence of detectable postural effects under the current methods, sample size, and exposure parameters, rather than as definitive evidence of no biological effect. Consequently, the possibility of Type II error should be considered when interpreting the absence of detectable exposure-related effects.

In addition, because this exploratory pilot study was not formally powered for definitive hypothesis testing, the possibility that subtle exposure-related effects remained undetected cannot be excluded. Accordingly, both the frequentist and Bayesian findings should be interpreted as preliminary and hypothesis-generating rather than confirmatory.

Overall, the Bayesian analyses suggest that moderate or large exposure-related effects are unlikely, while subtle effects cannot be completely ruled out.

The measured exposure levels (0.0030 W/m^2^; 1.5 V/m) were far below international safety limits (e.g., ICNIRP/IEEE: 40 W/m^2^ at this frequency) [13,14], supporting the interpretation that detectable short-term postural disturbances were not observed under the tested conditions.

The null exposure-related findings are also biologically plausible given the shallow penetration depth and low energy deposition at 3.6 GHz, which makes direct interaction with deep vestibular or proprioceptive structures unlikely. However, further research is needed to examine longer and cumulative exposure durations, repeated exposure patterns, higher but still compliant exposure intensities, different frequency bands, including higher mm Wave bands, and potentially sensitive populations.

Beyond the short-term outcomes examined here, longer or repeated RF-EMF exposure may induce adaptive biological responses, including alterations in oxidative stress regulation or physiological stress tolerance, which were beyond the scope of the present pilot study.

Although the present study did not directly assess molecular or biochemical outcomes, prior RF-EMF research suggests that oxidative stress pathways, regulation of reactive oxygen species, alterations in membrane potential, and related adaptive biological processes may contribute to longer-term physiological responses [71,75,76,77,78,79,80].

It also remains possible that transient neuromotor or vestibular effects occurring only during active exposure were not captured by the post-exposure assessment.

### 4.6. Limitations and Future Research

This pilot study provides initial insight into whether short-term 5G mobile phone EM exposure at 3.6 GHz influences static or dynamic postural balance. Although the study offers a valuable methodological foundation, several limitations should be acknowledged to guide interpretation and inform future research.

An additional limitation relates to exposure characterization. Although real-time EMF measurements were performed using the Narda EMR-300, this device is a broadband field meter and cannot definitively isolate individual frequency bands or fully exclude transient LTE handovers during real-world network operation. Consequently, the exposure condition in this study should be interpreted as a predominantly mid-band (~3.6 GHz) 5G mobile-phone exposure environment rather than a spectrally isolated 5G signal.

Furthermore, background static magnetic fields were not directly measured in the present study. Although the primary focus was on real-time RF-EMF exposure characterization associated with MP operation, static magnetic fields may potentially influence biological sensitivity to RF-EMF exposure and should therefore be considered in future experimental protocols.

A key limitation relates to the use of a single 5G device model, the Samsung Galaxy A13 (SM-A136B). While this ensured controlled and consistent exposure conditions, different phone models vary in antenna architecture, SAR levels, output power, and adaptive transmission behavior. These device-specific factors may meaningfully alter local exposure characteristics around the head and vestibular or proprioceptive sensory regions. As a result, findings derived from one handset may not generalize across the increasingly diverse ecosystem of 5G-enabled phones.

Another important limitation is the modest sample size, which is typical of exploratory pilot studies. Although the sample was adequate to test procedural feasibility, the limited statistical power means that subtle or inter-individual effects may have remained undetected. It is also possible that potential subgroup-specific responses, including sex-related or age-related differences in susceptibility to RF-EMF exposure, may have been masked within the overall group-level analyses.

Some Bayesian analyses returned Bayes factors close to 1, indicating inconclusive evidence rather than strong confirmation of either the null or alternative hypothesis. This uncertainty underscores that the current findings should be interpreted cautiously and highlights the need for larger, pre-registered trials.

Another limitation relates to the timing of the balance assessment. Because active EMFs interfered with the NeuroCom Balance Master sensors during pilot testing, all balance assessments were conducted immediately after exposure rather than during active mobile-phone operation. Consequently, the present study could not determine whether transient neuromotor or vestibular effects may occur exclusively during active RF-EMF exposure and rapidly dissipate following exposure cessation. Future studies should therefore develop safe real-time assessment protocols capable of measuring balance performance during active exposure without compromising sensor accuracy.

Another limitation is the short duration of exposure, limited to 5 to 20 min. Consequently, the present study was not designed to assess potential cumulative, adaptive, or longer-term biological responses associated with repeated RF-EMF exposure.

While these durations reflect typical short-term everyday MP use, they may not be sufficient to induce measurable physiological changes in balance networks. Balance control arises from multisensory integration spanning visual, vestibular, and proprioceptive systems; these mechanisms may require longer, repeated, or cumulative exposures before measurable alterations emerge. Therefore, the null findings should not be overinterpreted as definitive evidence of no detectable exposure-related effect under longer or repeated exposure scenarios.

The study sample comprised healthy adults, limiting generalizability to other populations. Populations such as older adults, younger individuals, or people with vestibular pathology, neuropathy, visual deficits, neurological conditions, or pre-existing postural instability may be more susceptible to subtle perturbations from EMF exposure. Likewise, developmental and hormonal factors, including known sex-related differences in vestibular and sensorimotor integration, were not examined and warrant investigation in larger cohorts. Hormonal fluctuations and known sex-dependent variability in balance performance may influence susceptibility to EMF-related effects, yet the present sample size was insufficient for stratified analyses. Similarly, the sample size was not adequately powered to detect potential age-related subgroup differences, and future studies with larger and more diverse cohorts are needed to examine demographic-specific susceptibility to RF-EMF exposure.

Future studies should recruit larger, age-diverse, and sex-balanced cohorts to better capture potential demographic and biological influences.

Exposure was also restricted to mid-band 5G at 3.6 GHz. Therefore, the findings of the present study should not be generalized to all 5G frequency bands or exposure environments.

It remains unknown whether balance outcomes would differ under low-band 5G (<1 GHz) or high-band millimetre-wave exposure, such as approximately 26 GHz, which exhibit different tissue penetration depths and absorption characteristics. Controlled laboratory studies across multiple frequency bands are essential to build a more comprehensive exposure–response profile.

Finally, although real-time EM measurements confirmed that the power density used in this study (0.0030 W/m^2^) was far below ICNIRP/IEEE public safety limits (40 W/m^2^), this very low intensity may partly account for the absence of measurable effects. It remains unclear whether higher, yet still compliant, exposure levels or longer-duration, environmentally realistic exposure patterns could influence multisensory postural systems. This represents an important direction for future research.

Direct near-field SAR measurement or participant-specific absorbed-dose modelling was not performed, limiting precise tissue-level dosimetry interpretation of the exposure conditions.

Taken together, these limitations may have reduced the sensitivity of the study to detect subtle, transient, cumulative, or subgroup-specific effects. Future research should therefore include larger and more diverse cohorts, multiple handset models, different 5G frequency bands, longer and repeated exposure durations, higher but still compliant exposure intensities, and safe real-time sensor-based assessment during active exposure.

### 4.7. Suggestions for Future Research

To advance this field, future research should follow a more structured and hypothesis-driven approach. Three interconnected priorities are particularly important.

First, future studies should systematically characterize exposure parameters and dose–response relationships. This research should test whether specific physical characteristics of RF-EMF exposure, including frequency band, exposure intensity, exposure duration, and repeated exposure patterns, influence biological or behavioral outcomes. Rather than relying on a single exposure protocol, controlled studies should compare different 5G frequency bands, including low-band and millimeter-wave spectra, which differ in propagation, tissue penetration, and absorption characteristics. Studies should also vary exposure intensity and duration to establish clearer dose–response relationships and assess whether cumulative or repeated exposure produces effects that are not detectable after brief exposure. In addition, longer and more ecologically valid behavioral contexts should be considered to determine whether effects emerge under conditions that more closely resemble real-world MP use.

Second, future research should determine whether population sensitivity modifies susceptibility to RF-EMF-related balance effects. Larger and more diverse samples are needed to permit robust subgroup analyses. Particular attention should be given to potentially vulnerable populations, including older adults and individuals with vestibular, neurological, proprioceptive, visual, or balance impairments. Demographic and biological variables, including age-related changes and sex-specific factors, should also be formally examined, as these may influence physiological responses to exposure and postural control performance.

Third, methodological and mechanistic precision should be improved. A key priority is the development of safe real-time assessment protocols that allow balance and neurophysiological function to be measured during active RF-EMF exposure. Such approaches would help capture transient or rapidly dissipating effects that may be missed by post-exposure testing. Future studies should also integrate high-resolution posturography with vestibular and ocular motor testing to identify which components of the balance system may be most sensitive to exposure. Finally, computational dosimetry and multimodal physiological monitoring, such as EEG or pupillometry, could help relate external exposure conditions to internal dose estimates and central nervous system activity, thereby clarifying potential mechanisms.

## 5. Conclusions

This sham-controlled pilot study found no detectable evidence that short-term 5- and 20-min low-intensity mid-band 5G mobile phone EM exposure at 3.6 GHz measurably altered static or dynamic postural control in healthy adults under the tested conditions. Across multiple instrumented balance domains, observed pre–post changes were small, exposure-independent, and most likely reflected task familiarization rather than exposure-related postural disruption. Bayesian analyses generally supported the absence of detectable exposure-related interaction effects, although subtle effects cannot be completely excluded due to the exploratory pilot nature of the study and modest sample size.

Overall, the findings provide preliminary evidence that brief predominantly mid-band 5G mobile phone EM exposure did not produce measurable postural disturbances in this cohort under the specific experimental conditions tested. These results should be interpreted cautiously and require confirmation in larger, adequately powered studies examining longer and repeated exposures, different frequency bands, and potentially sensitive populations.

## Figures and Tables

**Table 1:** Descriptive statistics (mean ± SD) for log-transformed unilateral stance (US) outcome measures across exposure conditions.

Outcome Measure	5 Min (Mean ± SD)	20 Min (Mean ± SD)	Sham (Mean ± SD)
Eyes Open (EO)	2.263 ± 0.089	1.796 ± 0.448	2.275 ± 0.107
Eyes Closed (EC)	2.255 ± 0.166	2.037 ± 0.179	2.339 ± 0.230

Data are presented as mean ± SD of transformed pooled outcome values across pre- and post-exposure assessments (*n* = 19 per condition). EO: eyes open; EC: eyes closed.

**Table 2:** Descriptive statistics (mean ± SD) for log-transformed rhythmic weight shift (RWS) outcome measures across exposure conditions.

Outcome Measure	5 Min (Mean ± SD)	20 Min (Mean ± SD)	Sham (Mean ± SD)
Left/Right (Comp, O-AV)	1.724 ± 0.016	1.749 ± 0.004	1.763 ± 0.041
Left/Right (Comp, DCL)	4.378 ± 0.014	4.406 ± 0.004	4.394 ± 0.011
Front/Back (Comp, O-AV)	1.364 ± 0.017	1.406 ± 0.052	1.313 ± 0.024
Front/Back (Comp, DCL)	4.370 ± 0.013	4.389 ± 0.002	4.340 ± 0.023

Data are presented as mean ± SD of transformed pooled outcome values across pre- and post-exposure assessments (*n* = 19 per condition). O-AV: on-axis velocity; DCL: directional control.

**Table 3:** Descriptive statistics (mean ± SD) for log-transformed limits of stability (LOS) outcome measures across exposure conditions.

Outcome Measure	5 Min (Mean ± SD)	20 Min (Mean ± SD)	Sham (Mean ± SD)
RT-Composite	−0.222 ± 0.021	−0.372 ± 0.050	−0.260 ± 0.030
EPE-Composite	4.334 ± 0.034	4.367 ± 0.041	4.261 ± 0.061
MXE-Composite	4.486 ± 0.009	4.501 ± 0.019	4.444 ± 0.029
DCL-Composite	4.357 ± 0.029	4.332 ± 0.042	4.294 ± 0.017
MVL-Composite	1.606 ± 0.008	1.747 ± 0.040	1.571 ± 0.008

Data are presented as mean ± SD of transformed pooled outcome values across pre- and post-exposure assessments (*n* = 19 per condition). RT: reaction time; EPE: endpoint excursion; MXE: maximum excursion; DCL: directional control; MVL: movement velocity.

## Data Availability

Data supporting the findings of this study, including de-identified datasets and analysis outputs, may be available from the corresponding author upon reasonable request, subject to Monash University Human Research Ethics Committee approval, participant consent restrictions, and institutional data-sharing requirements.
